# DKC1 enhances angiogenesis by promoting HIF-1α transcription and facilitates metastasis in colorectal cancer

**DOI:** 10.1038/s41416-019-0695-z

**Published:** 2019-12-20

**Authors:** Pingfu Hou, Peicong Shi, Tao Jiang, Hang Yin, Sufang Chu, Meilin Shi, Jin Bai, Jun Song

**Affiliations:** 1 Cancer Institute, Xuzhou Medical University, Xuzhou, Jiangsu China; 2 Center of Clinical Oncology, Affiliated Hospital of Xuzhou Medical University, Xuzhou, Jiangsu China; 3 Department of General Surgery, Affiliated Hospital of Xuzhou Medical University, Xuzhou, Jiangsu China; 4 School of Medical Imaging, Xuzhou Medical University, Xuzhou, Jiangsu China

**Keywords:** Oncogenes, Colorectal cancer

## Abstract

**Background:**

Dyskeratosis congenita 1 (DKC1) is dysregulated in several cancers. However, the expression and function of DKC1 in colorectal cancer (CRC) is rarely reported.

**Methods:**

Tissue microarrays (TAMs) including 411 cases of CRC tissues and corresponding paracancerous tissues were used to examine the DKC1 expression. The correlations between the DKC1 expression and clinicopathological or survival characters were further analysed. The functions and molecular mechanism of DKC1 in CRC were investigated through a series of in vitro and in vivo experiments.

**Results:**

The result showed that DKC1 expression was increased in CRC tissues. Increased DKC1 expression was associated with high grade of TNM stage, additional lymph node metastasis, and poor prognosis of patients with CRC. Multivariate COX analysis indicated that DKC1 can act as an independent prognostic factor for patients with CRC. DKC1 also facilitated the CRC angiogenesis and metastasis by increasing HIF-1α and VEGF expression levels. Chromatin immunoprecipitation assay demonstrated that DKC1 facilitated HIF-1α expression by regulating HIF-1α promoter activity.

**Conclusion:**

DKC1 appears to regulate CRC angiogenesis and metastasis through directly activating HIF-1α transcription. DKC1 can serve as an accurate indicator in predicting the prognosis of patients with CRC and act as a potential therapeutic target for CRC.

## Introduction

Colorectal cancer (CRC) is one of the most common malignancies worldwide. The incidence rate of CRC is the third highest in human tumours, and the mortality rate ranks second highest in malignant tumours.^1^ Most patients with CRC are already in the advanced stage of initial diagnosis, and the 5-year survival rate is low.^2,3^ Approximately 25% of patients with CRC have had liver metastases at the time of initial medical treatment.^4^ The data showed that ~45% of patients with CRC eventually died of this disease due to drug resistance and tumour metastasis after routine treatment and 80–90% of the liver metastases of patients with CRC have been unable to undergo radical resection of the tumour when they first consulted.^5,6^ Therefore, research on finding a potential biomarker for CRC is important for early CRC diagnosis and treatment.

Dyskeratosis congenita 1 (DKC1) gene was first discovered because its mutation caused dyskeratosis congenita (DC), which is an infrequent inherited syndrome, manifested as leukoplakia of oral, nail dystrophy, and unnatural reticulate skin pigmentation.^7,8^ Research has shown that the occurrence of DC can increase the incidence of many diseases, such as aplastic anaemia, bone marrow failure syndromes (BMFSs), and pulmonary fibrosis. Dyskerin that is encoded by DKC1 is a kind of nucleolar protein that is located at Xq28.^9,10^ Dyskerin is an important part of telomerase ribonucleoprotein complex and has a major impact on the functional stability of telomerase ribonucleoprotein complex. This phenomenon is mainly due to the participation of DKC1 in the production of telomerase and telomerase reverse transcriptase.^11,12^ The telomere length of patients with DC is significantly shorter than those of normal people.^13,14^ DKC1 also plays an important role in the processing of H/ACA small nucleolar ribonucleoprotein and is required for normal ribosome biosynthesis.^15^

Hypoxia region is formed when malignant tumours proliferate rapidly. In adapting to hypoxic microenvironment, tumour cells activate a variety of signal pathways and derive a large number of regulatory factors, which promote tumour tolerance to hypoxia and the tumour’s ability to metastasise as well as reducing sensitivity to radiotherapy and chemotherapy.^16,17^ Hypoxia-inducible factor 1 (HIF-1) is a transcription factor activated by hypoxia. HIF-1 consists of two subunits, that is, α and β.^18^ HIF-1α subunit can be regulated by O concentration, and its expression is positively correlated with the degree of hypoxia.^19,20^ HIF-1α also plays a vital role in tumour metastasis, proliferation, and angiogenesis.^21–23^ The hypoxic state of solid tumours becomes serious as malignant tumours continue to grow, which increases HIF-1α expression. HIF-1α overexpression further induces the expression of its downstream target gene VEGF and participates in tumour angiogenesis and metastasis.^24^ In CRC, HIF-1α expression is abnormally increased and plays an important role in the malignant progression of CRC.^25^

DKC1 is highly expressed in a variety of cancers.^26^ In neuroblastoma and renal cancer cells, DKC1 inhibition slows tumour growth and reduces tumour’s metastatic capacity. DKC1 upregulation is associated with the poor prognosis of patients with prostate cancer,^27^ hepatocellular carcinoma,^28^ and neuroblastoma.^29^ Meanwhile, DKC1 acts as a tumour suppressor and inhibits the development of pituitary tumours by downregulating the translational level of p27.^30^ In this study, we will explore the role of DKC1 in CRC progression.

## Methods

### Patients and specimens

The total of 8 pairs of fresh colorectal cancer tissues and corresponding paracancerous tissues were obtained directly from the operating room of Affiliated Hospital of Xuzhou Medical University. The tissues microarrays (TAMs) was composed of 411 CRC paraffin-embedded tissues and paired paracancerous tissues were obtained from the Pathology Department of Affiliated Hospital of Xuzhou Medical University from April 2010 to February 2015. The patients’ clinicopathological characteristics including age, sex, lymph node metastasis, tumour-node-metastasis (TNM) stage, differentiation, tumour diameter, distant metastasis as well as depth of invasion were also gained from the Pathology Department of Affiliated Hospital of Xuzhou Medical University.

### immunohistochemistry (IHC)

The CRC tissues and corresponding paracancerous tissues which were paraffin-embedded were punched to 1.5 mm diameter cores. The standard protocol for immunostaining of the TMAs was described previously.^31^ According to the streptavidin-peroxidase (Sp) method using a standard Sp Kit (Zhongshan biotech, Beijing, China). The slides were dewaxed at 65 °C for 2 h and then xylene was used to wash the slides for 20 min. The tissues were then rehydrated by washing the slides for 5-min each with 100%, 95%, 80%, 75%, 50% ethanol and finally with distilled water. The slides were then heated to 95 °C for 30 min in 10 mmol/L sodium citrate (pH 6.0) for antigen retrieval and then treated with 3% hydrogen peroxide for 1 h to block the endogenous peroxidase activity. Then subsequently the slides were incubated with specific antibodies at 4 °C overnight, and 3, 3′-diaminobenzidine (DAB; Zhongshan Biotech, Beijing, China) was used to produce a brown precipitate. The polyclonal rabbit anti-DKC1 (1:100, ab64667; Abcam, Cambridge, MA, USA) was used as primary antibody incubation at 4 °C overnight. For others primary antibodies, anti-HIF-1α antibody was applied at 1:400 dilutions (rabbit polyclonal, ab51608, Abcam, USA), anti-VEGF antibody was applied at 1:100 dilutions (rabbit polyclonal, 19003-1-AP, Proteintech, USA), anti-CD31 antibody was applied at 1:100 dilutions (rabbit polyclonal, ab51608, Proteintech, USA), anti-Ki67 antibody was applied at 1:100 dilutions (rabbit polyclonal, Ab-AF0198, Affinity, USA). We used Phosphate-buffered saline (PBS) instead of prime antibody as a negative control in time of primary antibody incubation. All images were recorded by Olympus BX-51 light microscope.

### Assessment of immunohistochemistry

Two pathologists independently evaluated the DKC1 staining scores without being informed of the clinical data. They discussed each core of disagreement and finally reached a unified opinion. We evaluated the expression score of DKC1 in CRC tissues and corresponding normal tissues by evaluating immunoreactive score (IRS) which was calculated by multiplying the intensity of DKC1 staining and the percentage of DKC1 immune positive cells. The intensity of DKC1 immunostaining was graded as 0–3 (0, negative; 1, weak; 2, moderate; 3, strong). The percentage of immune positive cells was scored into 4 ranks: 1 is range from 0 to 25%, 2 is range from 26 to 50%, 3 is range from 51 to 75%, and 4 is range from 76 to 100%. On the basis of the IRS, the level of DKC1 expression was classified as low (IRS: 0, 1, 2, 3, 4, 6) and high (IRS: 8, 9, 12) expression.

### Cell lines and cell culture condition

The colon cell lines: FHC, LoVo, SW620, SW480, DLD1, and HCT116 cell lines were obtained from the Shanghai Institute of Biochemistry and Cell Biology, Chinese Academy of Science (Shanghai China). FHC, LoVo and DLD1 cells were cultured in RPMI-1640 medium while SW620, SW480 and HCT116 were cultured in DMEM medium containing 10% foetal bovine serum, 100 U/ml penicillin and 100 μg/ml streptomycin. All the cell lines were incubated in a 37 °C humidified incubator with 5% CO_2_.

### RNAi and stable cell lines construction

The siRNA specific for DKC1 (siDKC1) and scrambled siRNA were obtained from Shanghai GenePharma Co., Ltd. (Shanghai, China). The siRNA was transfected by using siLentFect^TM^ Lipid reagent (Bio-Rad Laboratories, Hercules, CA, USA) at a cell density of 30%. In each 60 x 15 mm cell culture dish, 40 µg DKC1 small interfering (si)RNA or negative control siRNA (Shanghai GenePharma Co., Ltd.) were transfected using 8 µl siLentFect Lipid Reagent. A total of 48 h after transfection, the cells were used for subsequent experimentation according to the manufacturer's protocol. The siRNA sequences are listed as follow:

siCtrl: 5’-UUCUCCGAACGUGUCACGUTTACGUGACACGUUCGGAGAATT-3’,

siDKC1#1: 5’-GCGGAUGCGGAAGUAAUUATTUAAUUACUUCCGCAUCCGCTT-3’,

siDKC1#2: 5’-CCGGCUGCACAAUGCUAUUTTAAUAGCAUUGUGCAGCCGGTT-3’,

The shRNA target sequence is listed as follow:

shDKC1: 5’-CCGGCUGCACAAUGCUAUU-3’.

The Plasmid of pLV-EGFP: T2A: Puro-EF1A-hDKC1 and was purchased from VectorBuilder lnc and the Plasmid of HIF-1α was a gift from Dr. Rui Chen (Capital Medical University). CRC cells were grown to approximately 50% confluency and then transiently transfected with plasmids using lipofectamine 2000 transfection reagent (Invitrogen) according to the manufacturer’s instructions. Twelve hours after transfection, the medium containing transfection reagents was removed, and the cells were incubated in fresh medium. The DKC1 stable knockdown HCT116 cell lines and control cells lines were structured by using lentivirus packing DKC1 shRNA and corresponding control vector (Shanghai GenePharma). The DKC1 knockdown HCT116 cells were then infected with HIF-1α overexpression lentivirus (GENECHEM) to establish HIF-1α rescue cell lines. These target cells were transfected with lentivirus for 48 h and then selected with 5 µg/ml puromycin (Santa Cruz Biotechnology, Inc., Dallas, TX, USA) for 2 weeks.

### Cell migration and invasion assays

Cell migration and invasion assays were performed as described previously by using the Transwell chambers.^32^ Matrigel (BD Biosciences, Mississauga, Canada) was uncovered or covered the Transwell filter inserts for cell migration assays or cell invasion assays respectively. 2 × 10^5^ cells resuspended with FBS-free medium were seeded in the top chamber and incubated at 37 °C with 5% CO_2_ for 12 or 24 h. Then fixed the cells across the membrane with 90% methanol for 20 min and stained with Crystal violet. The cells unable to traverse the membrane in top chamber were obliterated with cotton swabs simultaneously. The cells that had traversed the membrane were count by using inverted microscope.

### Tube formation assay

1 × 10^6^ DKC1 overexpression, knockdown, and HIF-1α rescue CRC cells along with the respective control cells were incubated with 2 ML fresh FBS-free medium in 60-mm plates for 1 day and then the treated medium was preserved. For tube formation assay, the 48-well plate coated with 180 µL Matrigel^TM^ (BD Biosciences) was kept in a 37 °C humidified incubator for 2 h to curdle the Matrigel. Then 4 × 10^4^ HUVECs cells suspended by 200 µL treated medium were seeded into the 48-well plate which was coated with Matrigel previously and cultured for 4 h. Inverted microscope was used to count the tubes formation.

### RNA isolation and Real time PCR

The total RNA extraction was performed by using Trizol reagent (Invitrogen) in accordance with the manufacturer’s instructions. After measuring RNA purity, the reverse transcription reaction was carried out (1 µg RNA) with PrimeScript™ RT reagent Kit along with the gDNA Eraser (Vazyme). Primer sequences are listed as follow:

5’-TGATGACCAGCAACTTGAGG-3’(forward) and

5’-CTGGGGCATGGTAAAAGAAA-3’(reverse) for HIF-1α,

5’-GAAGGACTTTACCTTCCAGGA-3’(forward) and

5’-ATGATTCTGCCCTCCTCCTTC-3’(reverse) for VEGF,

5’-CTTGATTCTGGAGCCAGTGTTCT-3’ (forward) and

5’-TTTGATCTTCACGCTACTTTTGTT-3’ (reverse) for P1,

5’-GACGGAGTCTCGCTACGTTC-3’ (forward) and

5’-GACAGGGTGGTTCCAGCTAC-3’ (reverse) for P2,

5’-TGTGCAATGCTACTTTGTTGGG-3’ (forward) and

5’-CCTTTGGCAACTTTGCAAGCTA-3’ (reverse) for P3,

5’-TCTGGTAAGGAAAGACCCCG-3’ (forward) and

5’-AAAAAGCAGACTTCGCCTCG-3’ (reverse) for P4,

5’-TTCTCTTTCCTCCGCCGCTA-3’ (forward) and

5’-CCAATCAGGAGGCGGTCAG-3’ (reverse) for P5,

5’-GTCGCTCGCCATTGGATCT-3’ (forward) and

5’-CTCCTGTCCCCTCAGACGA-3’ (reverse) for P6.

5’-AAGGTCGGAGTCAACGGATTTG-3’(forward) and

5’-CCATGGGTGGAATCATATTGGAA-3’(reverse) for GAPDH.

Real-time PCR was performed by ABI7500 qRT-PCR system thermal cycler (Vazyme Biotech, Nanjing, China) with SYBR Green PCR Master Mix in triplicate. GAPDH mRNA was chosen to be internal control. CT method was used to calculate the target mRNA levels, normalised to GAPDH.

### Western blot analysis

Western blot analysis was carried out as described previously.^33^ The rabbit anti-DKC1 (1:1500, ab64667, Abcam, USA), anti-HIF-1α (1:1000, ab51608, Abcam, USA), anti-VEGF (1:1000, 19003-1-AP, Proteintech, USA) and mouse anti-GAPDH (1:200000, 60004-1-Ig Proteintech, USA) were used for the primary antibodies incubation at 4 °C overnight. Horseradish peroxidase (HRP)-goat anti-rabbit, HRP-goat anti-mouse were used for secondary antibodies. The signals were identified using the Tanon 6600 Luminescent Imaging Workstation (Tanon Science & Technology Co., Ltd., Dalian, China). Densitometry was used to measure protein bands’ intensity.

### Dual-luciferase reporter assays

Dual-luciferase reporter assays was performed as described previously.^34^ HCT116 cells were seeded onto 24-well plates (5 × 10^4^ cells per well) and transfected with siRNA specific for DKC1 or non-specific control siRNA, HIF-1α promoter plasmid, renilla luciferase plasmid. Two days later, we measured the activities of both firefly luciferase and renilla luciferase according to the dual luciferase reporter assay system (Promega, Madison, WI, USA). The internal standard for transfection efficiency was normalised to renilla luciferase activity. The HIF-1α promoter region (−1800/+200) was cloned to the PGL4.20 plasmid (Promega).

### Chromatin immunoprecipitation (ChIP) assay

ChIP assay was carried out as described previously.^35^ Anti-DKC1 or negative control anti-IgG were incubated with ChIP dilution buffer containing the sheared DNA at 4 °C overnight rotationally. qRT-PCR was carried out to exaggerate the genomic region of DKC1 flanking the possible DKC1 binding sites.

### Subcutaneous tumour model and lung metastasis model in vivo

The animal experiments were approved by the Animal Care Committee of the Xuzhou Medical University, Xuzhou, China. The female BALB/c nude mice (6–8 weeks old) were purchased from Beijing Vital River Laboratory Animal Technology Co., Ltd. The female BALB/c nude mice were fed under specific pathogen-free condition. Provide details of: The animals were maintained in a controlled environment with controlled temperature (~25 °C), humidity (50–70%) and (light, 07:00; dark, 22:00). The water and mouse feed were sterilised by uperisation and were freely available. The total of 20 female BALB/c nude mice were divided into two groups randomly: DKC1 knockdown and control group. DKC1 knockdown (5 × 10^6^) and control (5 × 10^6^) HCT116 cells were suspended with 200 μl PBS and were injected subcutaneously into the axilla. Six days later, the subcutaneous tumours can be observed by vision. Tumours volumes were measured by multiplying long diameter and wide diameter every two days. Two weeks later, all the mice were sacrificed by cervical dislocation in fume cupboard and the tumours were excised. Every tumour was weighed and fixed by 4% paraformaldehyde for further immunohistochemistry assay. Lung metastasis model was established by mice’s tail vein injection. Twenty-one female BALB/c nude mice (6–8 weeks old) were randomly divided into three groups: control, DKC1 knockdown and HIF-1α rescue based on DKC1 knockdown HCT116 cells. 2 × 10^6^ HCT116 cells suspended with 200 μl PBS were injected through the mice’s tail vein. Forty-five days later, all the mice were sacrificed by cervical dislocation in fume cupboard and the lungs were excised. Each lung was fixed by 4% paraformaldehyde for further H.E. staining and immunohistochemistry assay and metastatic nodules on the surface of each lung were counted.

### Cell proliferation assay

CCK-8 assay was performed to detect the cell proliferation using Cell Counting Kit-8 manufacturer's protocol (Dojindo) as described previously.^36^ Briefly, 4000 cells suspended with 200 μL complete medium were seeded into the 96-well plates and cultured. When the specified time was reached, 10 μL CCK-8 solution mixed with 100 μL medium was added into the 96-well plates and cultured for 2 h at 37 °C. The absorbance was measured at 450 nm.

### Cell cycle analysis

Cell cycle assay was performed as described previously.^37^ Cells were treated in serum-free medium for 12 h to synchronise cell cycle, then the complete medium was used to re-enter the cell into the cell cycle. Afterwards, cells were fixed with 70% ethanol at 4 °C for 12 h. The fixed cells were suspended with RNase A and then cells were mixed with propidium iodide (PI). Finally, flow cytometry (BD, FACSCantoTM II) was used to detect the cell cycle.

### Statistical analysis

The SPSS 20.0 was used for all statistical analysis. The difference between DKC1 staining of CRC tumours and its corresponding adjacent non-cancerous tissues were evaluated by paired Wilcoxon test. χ^2^-test was used to evaluate the relationship between the expression of DKC1 and clinicopathological features. Kaplan–Meier method and log-rank test were chosen to investigate the correlation between DKC1 expression and 5 years overall survival and disease-free survival. Univariate and multivariate Cox proportional hazards regression analysis were carried out to assess the crude hazard rations (HRs), adjusted HRs and 95% confidence interval (CI). Whether there is a difference between the two groups depends on the unpaired *t*-test statistically.

## Results

### DKC1 expression was significantly increased in CRC tissues and cell lines

**Fig. 1:**
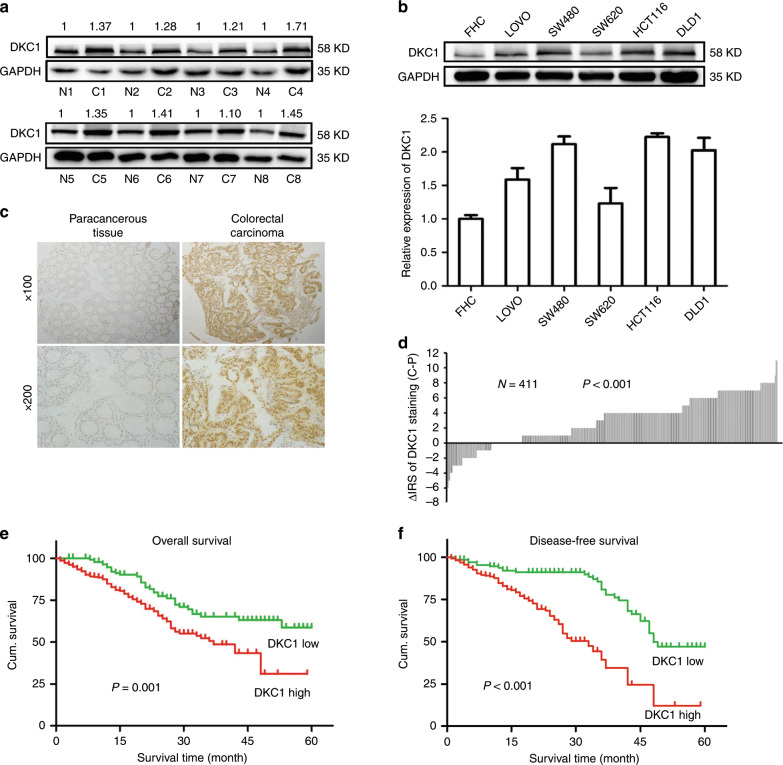
DKC1 Expression was upregulated in CRC and negatively associated with overall and disease-free survival in CRC. **a** Detection of DKC1 protein levels in 8 cancer tissues and paired normal colon tissues by western blot. **b** The expression level of DKC1 was detected by western blot in five CRC cell lines and a normal colon epithelial cell line (FHC). **c** Representative images of DKC1 immunohistochemical staining in TMAs were shown. Note: Top panel: original magnification, ×100; bottom panel: original magnification, ×200. **d** The distribution of the difference in staining intensities of DKC1 in CRC tissues compared with that in paired paracancerous tissues. C, CRC tissues; P, paired paracancerous colon tissues (*P* < 0.001, the paired Wilcoxon test). **e** High DKC1 expression related to greater favourable overall cumulative survival of RCC patients (*P* = 0.001, log-rank test). **f** High DKC1 expression related to greater favourable disease-free cumulative survival of RCC patients (*P* = 0.003, log-rank test). Data are shown as mean ± standard deviations. *P < 0.05, **P < 0.01, ***P < 0.001.

To determine whether DKC1 is involved in CRC development, we first assessed DKC1 protein expression in the tumour and paracancerous tissues of eight patients with cancer, its expression significantly increased in CRC tissues compared with normal tissues (Fig. 1a). Subsequently, Western blot analysis showed that DKC1 expression in five different CRC cell lines (LOVO, SW480, SW620, HCT116, and DLD1) was considerably upregulated compared with normal colon epithelial cell line FHC (Fig. 1b). To prove the findings above, we next used immunohistochemistry (IHC) to study DKC1 expression in tissue microarrays. The results showed that DKC1 was predominantly located in the nucleus (Fig. 1c). As shown in Table 1 and Fig. 1d, DKC1 IRS was conspicuously higher in 279 out of 411 (68%) CRC tissues and significantly lower in 324 out of 411 (79%) adjacent noncancerous tissue.

**Table 1:** Relationship between DKC1 expression and clinicopathological features of CRC patients.

Variables	Cases	DKC1 expression (*n* = 411 cases)		*P*^ a^
		Low (%)	High (%)	
All patients	411	132 (100)	279 (100)	
Age (years)				0.593
≤ 60	172	58 (44)	114 (41)	
>60	239	74 (56)	165 (59)	
Gender				0.292
Males	203	60 (45)	143 (51)	
Females	208	72 (55)	136 (49)	
Lymph node metastasis				0.013
N0	250	92 (70)	158 (57)	
N1/N2/N3	161	40 (30)	121 (43)	
TNM stage				<0.001
I		45 (34)	62 (22)	
II		58 (44)	86 (31)	
III		28 (21)	125 (45)	
IV		1 (1)	6 (2)	
Differentiation^b^				0.108
Poor	99	25 (19)	74 (27)	
Moderate/high	307	105 (81)	202 (73)	
Tumour diameter				0.632
≤5 cm	203	60 (45)	143 (51)	
>5 cm	208	72 (55)	136 (49)	
Distant metastasis				0.003
M0	379	129 (98)	250 (90)	
M1	32	3 (2)	29 (10)	
Depth of invasion				0.507
T1/T2	143	49 (37)	94 (34)	
T3/T4	268	83 (63)	185 (66)	

^a^Two-sided Fisher’s exact tests

^b^The type of differentiation of cancer in five patients cannot be assessed

### High DKC1 expression was correlated with clinicopathologic characteristics and poor survival in patients with CRC

We used Fisher’s exact test to investigate the relationship between DKC1 expression and clinicopathological parameters in patients with CRC. The expression of DKC1 is also high in patients with high TNM stage (*P* < 0.001, χ^2^-test). Our data revaluated that the upregulated DKC1 expression was also dramatically positively associated with the distant metastasis (*P* = 0.003, χ^2^-test). By contrast, the DKC1 expression and patients’ age, gender, differentiation, tumour diameter, or invasion depth had no relationship (Table 1).

We used Kaplan–Meier analysis and log-rank test to evaluate the function of DKC1 in patients with CRC. Our data demonstrated that the patients who showed upregulated DKC1 signals corresponded with poorer 5-year overall (*P* = 0.001) and disease-specific cumulative survival (*P* < 0.001) than those with few DKC1 signals (Fig. 1e, f).

Next, we used univariate and multivariate COX analysis model to explore whether DKC1 expression was an independent prognostic factor for patients with CRC. The univariate COX regression analysis showed that DKC1 expression, TNM stage, and lymph node metastasis were all the important prognostic factors for the overall survival of patients with CRC. At the same time, our data showed that they combined with the tumour differentiate were also the significant prognostic factors for the disease-specific survival of patients with CRC performed by the univariate COX analysis (Supplementary Table S1). We also used multivariate COX regression analysis to confirm that DKC1 expression and TNM stage were the vital factors for the overall and disease-specific survival of patients with CRC (Supplementary Table S2).

### DKC1 promoted human CRC cell migration, invasion, and angiogenesis in vitro

**Fig. 2:**
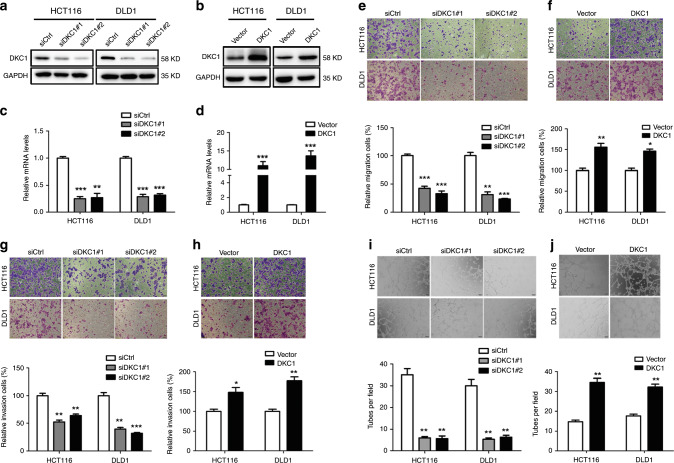
DKC1 promoted angiogenesis, migration and invasion of CRC cells. : (**a, b**) Western blot was used to test the DKC1 expression in HCT116 and DLD1 cells with DKC1 knockdown (siRNA) and DKC1 overexpression (pLV-EGFP-DKC1). (**c**, **d**) Real-time PCR was used to test the DKC1 expression in HCT116 and DLD1 cells with DKC1 knockdown(siRNA) and DKC1 overexpression (pLV-EGFP-DKC1). (**e**–**h**) Effects of DKC1 knockdown or overexpression on the migration and invasion in colon cancer cells. (**i**, **j**). Effects of DKC1on tube formation. Numbers of complete tubular structures formed by HUVECs were counted for DKC1 knockdown or overexpression in HCT116 and DLD1 cells. Scale Bar: 50μm. Data are presented as the means ± SD for experiments in triplicate. Data are shown as mean ± standard deviations. *P < 0.05, **P < 0.01, ***P < 0.001.

According to our previous research, CRC cohort suggested that DKC1 expression was closely correlated with CRC metastasis. To elucidate the role of the DKC1 in colon cancer cells, we investigated its role in colon cancer cell migration and invasion. We transfected control small interfering RNAs and specific siRNAs targeting DKC1 (i.e., siDKC1#1 and siDKC1#2) or pLV-EGFP-control and pLV-EGFP-DKC1 transiently in HCT116 and DLD1 cells, respectively. After 48 h, DKC1 protein and mRNA levels were knocked down in the two colon cancer cells (Fig. 2a, c, respectively). Meanwhile, 24 h later, DKC1 protein and mRNA expression were overexpressed in HCT116 and DLD1 cells (Fig. 2b, d, respectively). Transwell assay showed that the capacity of tumour cell migration and invasion was dramatically reduced when DKC1 decreased in HCT116 and DLD1 cell lines (Fig. 2e, g). By contrast, when DKC1 was overexpressed, the amount of cell migration and invasion through the artificial extracellular matrix significantly increased in HCT116 and DLD1 cells (Fig. 2f, h).

Previous studies showed that angiogenesis played a crucial role when tumours metastasised. To investigate whether DKC1 affects the ability of angiogenesis, we knocked down and overexpressed DKC1 in HCT116 and DLD1, and then the medium without foetal bovine serum was used to culture the processed cells for 24 h. Afterwards, we cultured HUVECs on Matrigel with the medium used above. The ability of HUVECs that were cultured with the medium collected from the DKC1 knocked down cells to form tubular structure was decreased compared with the corresponding controls (Fig. 2i). By contrast, when we used the medium that was collected from the cells, DKC1 was overexpressed to culture HUVECs, and the number of the tubes in which the HUVECs formed significantly increased compared with the control group in vitro (Fig. 2j). In addition, we also showed that DKC1 facilitates cell proliferation (Supplementary Fig. S1A), but DKC1 has little effect on cell cycle (Supplementary Fig. S1B).

### DKC1 facilitated CRC cell migration, invasion, and angiogenesis by increasing HIF-1α expression

**Fig. 3:**
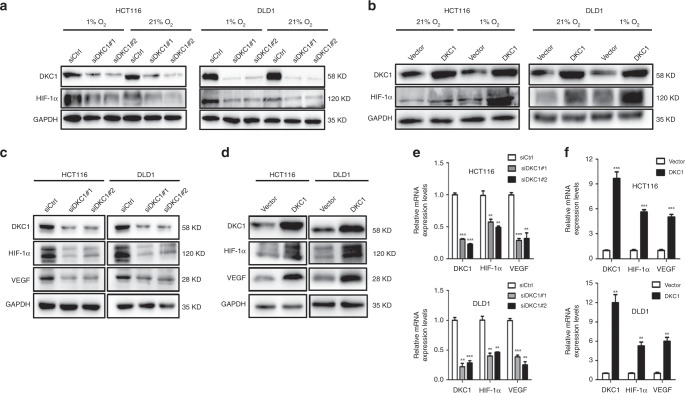
DKC1 regulated the expression of HIF-1α and VEGF. **a**, **b** Western blot of HIF-1α expression in DKC1 knockdown or overexpression HCT116 and DLD1 cells grown under normoxia or hypoxia (1% O_2_) for 4 h. GAPDH was used as a loading control. **c**, **d** Western blot of HIF-1α and VEGF expression in DKC1 knockdown or overexpression HCT116 and DLD1 cells. **e**, **f** Real-time PCR of HIF-1α and VEGF mRNA level in DKC1 knockdown or overexpression HCT116 and DLD1 cells. Values were normalised against GAPDH from three independent experiments and are presented as means ± SD. Data are shown as mean ± standard deviations. *P < 0.05, **P < 0.01, ***P < 0.001.

HIF-1α plays an important role in tumour metastasis and angiogenesis.^38^ Our data showed that DKC1 may have effects on CRC cell migration, invasion, and angiogenesis. Then, we further study whether DKC1 directly regulates HIF-1α expression and its downstream target expression. Western blot analysis showed that DKC1 downregulation in HCT116 and DLD1 cells caused the inhibition of HIF-1α in normoxic (21% O_2_) or hypoxic (1% O_2_) environment. Meanwhile, when DKC1 was overexpressed, HIF-1α protein levels were also significantly increased (Fig. 3a, b). HIF-1α-VEGF pathway is a classical signal pathway that can regulate tumour angiogenesis and metastasis.^24^ To explore whether DKC1 has an effect on HIF-1α-VEGF pathway further, we performed Western blot analysis and real-time PCR to verify the HIF-1α and VEGF protein and mRNA expression levels when DKC1 was knockdown or overexpression. Our data indicated that HIF-1α and VEGF protein and mRNA expression were also positively regulated by DKC1 (Fig. 3c–f).

### DKC1 regulated HIF-1α expression independently of interfering its protein stability

**Fig. 4:**
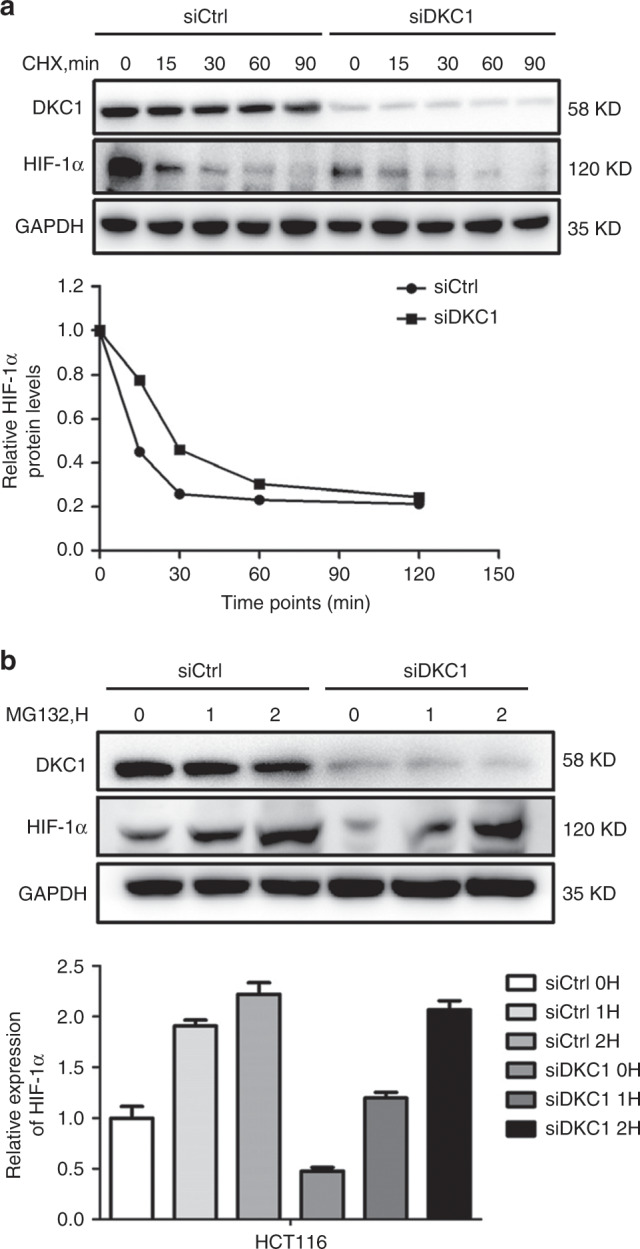
DKC1 regulated the expression of HIF-1α independently of interfering its protein stability. **a** Top panel: western blot showed HIF-1α protein decay in HCT116 cells ± DKC1 KD at the indicated time points (0, 15, 30, 60, 90 min) after cycloheximide (CHX) addition under 1% O_2_. GAPDH was used as a loading control. bottom panel: Graphical representation of HIF-1α protein levels from top panel based on Grayscale analysis. Half-lives are shown under the curves. **b** Western blot showed HIF-1α protein in HCT116 cells ± DKC1 KD at the indicated time points after MG132 addition. GAPDH was used a loading control.

To investigate whether DKC1 regulated HIF-1α by affecting its protein stability, we barricaded the translation of HIF-1α by using cycloheximide (CHX), an inhibiting agent of protein, and collected the cells on schedule. The HIF-1α degradation rates stayed the same in the mass regardless of DKC1 expression in HCT116 cells (Fig. 4a). The previously synthesised HIF-1α protein has the same decay rate regardless of whether the DKC1 content is high or low. This phenomenon argued against DKC1 regulating HIF-1α protein stability. We also used the MG132, which is an inhibitor of proteasome, to test HIF-1α expression between control and DKC1 KD cells. As shown in Fig. 4b, HIF-1α in DKC1 KD cells was not restored to the same level as the control cells. MG-132 treatment abolished the downregulation of protein HIF1a levels in CRC cells with knockdown of DKC1. These two experiments together illustrated that DKC1 regulated HIF-1α expression independently of interfering its protein stability.

### DKC1 promotes CRC migration, invasion and angiogenesis through transcriptional regulating HIF-1α

**Fig. 5:**
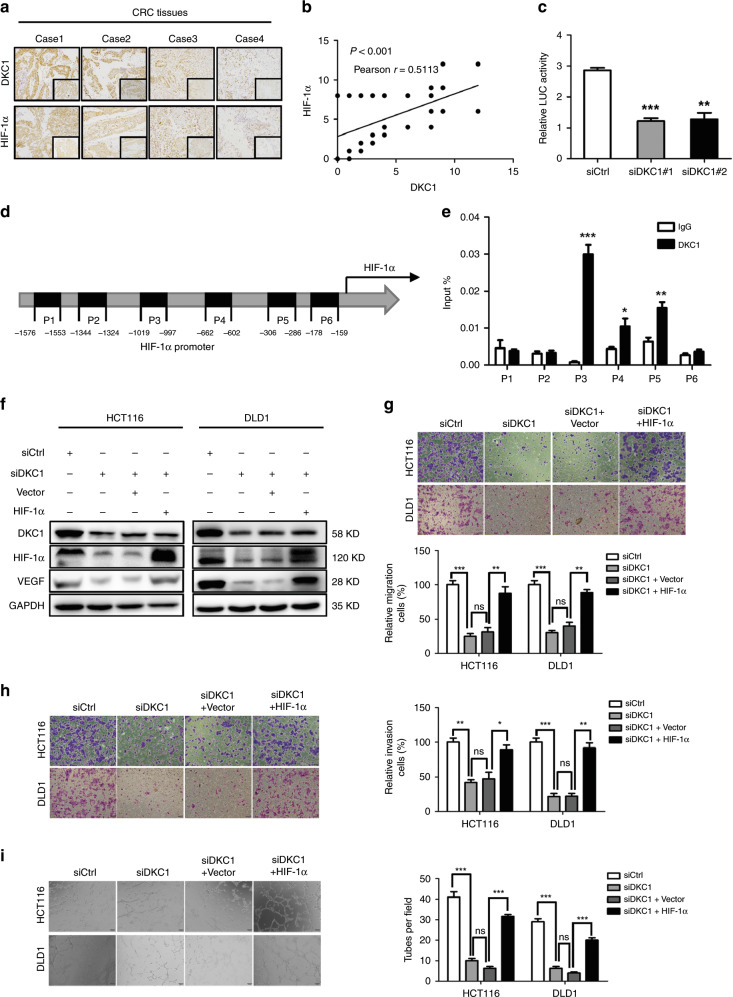
DKC1 facilitated the transcription of HIF-1α. **a** Representative images of DKC1 and HIF-1α immunohistochemical staining in TMAs were shown. **b** Analysis of the relationship between DKC1 expression and HIF-1α levels in CRC patient tissues from TMAs. **c** The HIF-1α promoter (−1800/+200) activity was increased by DKC1 knockdown in HCT116 cells. **d** Full sequence of the human HIF-1α promoter (−1800 bp-+200 bp) . P1-P6 showed the regions of HIF-1α promoter detected by the paired primers. **e** ChIP-qRT-PCR analysis of DKC1 binding at P1, P2, P3, P4, P5 and P6. **f** Western blot analysis revealed that the VEGF expression in DKC1‑knockdown HCT116 and DLD1 cells was significantly rescued by HIF-1α plasmid when compared with the corresponding controls. **g**–**i** The inhibition of migration, invasion and angiogenesis caused by DKC1 knockdown was regressed by HIF-1α overexpression in HCT116 and DLD1 cell lines. Data are shown as mean ± standard deviations from three independent experiments. *P < 0.05, **P < 0.01, ***P < 0.001.

DKC1 regulated somatic cell reprogramming and stem cell maintenance by interacting with OCT4 and SOX2 to be recruited into the promoter of the target gene. We speculated whether DKC1 regulated HIF-1α expression at the transcriptional level by acting on HIF-1α promoter. To confirm the relationship between DKC1 and HIF-1α further, we performed IHC to examine HIF-1α expression compared with DKC1 expression in tissue microarrays. As shown in Fig. 5a, b, a positive correlation was observed between DKC1 and HIF-1α expression levels (Pearson’s, r = 0.5113, *P* < 0.001). Dual-luciferase reporter assay showed that the HIF-1α promoter’s transcriptional activity was significantly decreased when DKC1 was knockdown (Fig. 5c). Subsequently, HIF-1α promoter region was described, and we designed six fragments that were the potential binding regions in the HIF-1α promoter (Fig. 5d). Next, chromatin immunoprecipitation (ChIP) assay was carried out to investigate the fragment of HIF-1α promoter that can be bonded together with DKC1. The ChIP-qRT-PCR results indicated that DKC1 was mainly bound to the P3-P5 binding regions of the HIF-1α promoter (Fig. 5e).

To assess the role of HIF-1α in DKC1 induced CRC malignant characters, we performed HIF-1α rescue assays. The plasmids that can overexpress HIF-1α and two independent siRNAs targeting DKC1 were co-transfected in HCT116 and DLD1 cells. The results showed that HIF-1α rescue can remarkably save the VEGF expression (Fig. 5f). Meanwhile, the cells’ capacities with regard to migration, invasion, and angiogenesis were also reversed by HIF-1α rescue (Fig. 5g–i).

### DKC1 facilitated CRC cell angiogenesis, metastasis, and proliferation in vivo

**Fig. 6:**
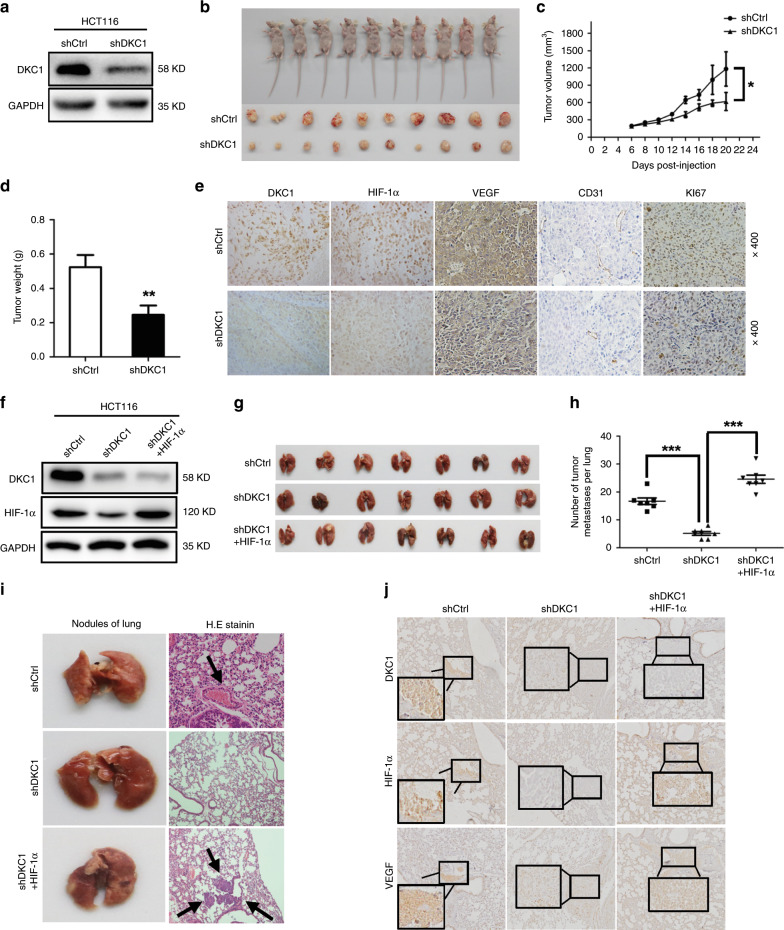
DKC1 facilitated the angiogenesis, metastasis and proliferation of CRC cells in vivo. **a** DKC1 knockdown in HCT116 stable cell line was confirmed at the protein level by western blotting. **b** General observation of the subcutaneous tumours in nude mice formed by HCT116 cells transfected with shDKC1 and shCtrl lentivirus (*n* = 10 in each group) and twenty days after injection, the mice were killed, and the xenograft tumours were collected. **c** Tumour volumes of xenografts in nude mice. Xenografts volumes were calculated with the following formula: V = (L × W2)/2. **d** Weight of the xenograft tumours was analysed. **e** The tumour sections were performed immunochemistry staining by antibody against DKC1, HIF-1α, VEGF, CD31 and Ki67; representative images were shown (×400 magnification). **f** Western blot was used to test the DKC1 and HIF-1α expression in HCT116 cells with DKC1 knockdown(shRNA) and HIF-1α overexpression (shRNA). **g** HCT116 cells with DKC1 stable knockdown or HIF-1α overexpression or control cells were injected into BALB/c nude mice’s tail vein. 45 days after injection, all the mice were killed, and the lungs were excised. **h** The number of metastatic nodules of lung was counted. **i** H.E staining was performed to further confirm the condition of metastatic nodules of lung. **j** The lungs were performed immunochemistry staining by antibody against DKC1, HIF-1α and VEGF; representative images were shown (×400 magnification). *P < 0.05, **P < 0.01, ***P < 0.001.

Hence, DKC1 has positive influences on colon cell angiogenesis, metastasis, and proliferation in vitro. Then, we established stable cell lines that DKC1 was knocked down in HCT116 cells for further research. Western blot analysis showed that DKC1 was knocked down in the stable cell lines (Fig. 6a). We injected the stable DKC1-KD and control cells that were mixed with Matrigel into BALB/c nude mice subcutaneously. After 6 days, we measured the maximum length and width of the subcutaneous tumours every second day when it can be significantly observed (Fig. 6b). After 2 weeks, we resected the subcutaneous tumours and measured their weight and volume. Our data exhibited a significant decrease in the weight and volume of the tumours with DKC1 stable knockdown cells compared with the control cells (Fig. 6c, d). IHC assay showed that the DKC1 knockdown xenograft tumour HIF-1α and VEGF protein expression was decreased compared with the control group (Fig. 6e). CD31 for assessing tumour angiogenesis and Ki67 for assessing tumour proliferation were also reduced in DKC1 knockdown xenograft tumour (Fig. 6e). We established stable HIF1α-overexpression cell lines on the basis of stable DKC1-knockdown cells. Then, we injected HCT116 cells with DKC1 stable knockdown or HIF-1α rescue or control cells into BALB/c nude mice tail vein. After 45 days, all mice were sacrificed, and the lungs were excised. Visual analysis indicated that the number of tumour metastases were significantly reversed by HIF-1α rescue (Fig. 6f–h). This result was further confirmed by lung HE staining (Fig. 6i). IHC assay showed that the VEGF expression was upregulated by HIF-1α rescue (Fig. 6j).

## Discussion

With the advancement of neoadjuvant chemoradiation,^39^ surgical procedures,^40^ and endoscopic treatment^41^ for CRC, some early diagnosed CRC treatments have achieved good therapeutic results, and the patient’s 5-year survival rate has improved. However, for many advanced CRCs, especially CRCs that have undergone distant metastasis, an effective treatment is still unavailable.

Tumour development is a multifactor, multistep complex process, including oncogene activation and tumour suppressor gene inactivation. DKC1 was first discovered because its mutation caused DC, which is associated with BMFSs and tumour susceptibility.^42^ For neuroblastoma, DKC1 expression inhibition slows down cell proliferation and inhibits xenograft tumour growth in nude mice.^29^ DKC1 knockdown inhibits P65 expression in the NF-κB pathway and reduces the proliferation and metastasis capacity of renal cancer cells.^43^ In the present study, DKC1 protein is highly expressed in CRC tissues compared with paracancerous tissues (Fig. 1a), and high DKC1 expression is associated with high grade of TNM stage and additional lymph node metastasis (Table 1). DKC1 upregulation is associated with poor prognosis in patients with prostate cancer, neuroblastoma, and hepatocellular carcinoma.^27–29^ These findings are consistent with our studies showing that upregulated DKC1 expression is correlated with poor 5-year overall and disease-specific cumulative survival in patients with CRC (Fig. 1e, f). Our studies showed that DKC1 serves as an independent prognostic factor for patients with CRC analysed by univariate and multivariate COX analysis model (Supplementary Tables S1 and S2).

Angiogenesis is one of the key factors in tumour invasion and metastasis.^44^ Tumour angiogenesis is an extremely complex process that generally involves the steps of vascular endothelial matrix degradation, endothelial cell migration, endothelial cell proliferation, endothelial cell tube branching to form vascular rings, and formation of a new basement membrane. When neovascularisation is formed inside the tumour tissue, it provides a large amount of nutrients to the tumour cells, thereby causing the cells to grow rapidly and infiltrate into the surrounding tissues, and has a tendency to metastasise.^45^ Our studies showed that HUVECs’ ability to form the tube was enhanced in conditioned medium collected from HCT116 cells and DLD1 cells overexpressing DKC1 (Fig. 2j). This phenomenon was further confirmed because when DKC1 was knockdown, the number of tubes was decreased compared with the control group (Fig. 2i). Transwell assays demonstrated that the trend of cell migration and invasion ability was consistent with the trend of angiogenic ability when DKC1 was overexpressed or knocked down (Fig. 2e–h).

VEGF is an important proangiogenic factor that provides optimal matrix for tumour cell growth and new capillary network by enhancing vascular permeability and extravasation of fibrinogen, and VEGF stimulates proliferation of vascular endothelial cells and promotes angiogenesis.^46^ Our results confirmed that DKC1 can positively regulate VEGF expression. HIF-1α-VEGF pathway is a classical signal pathway to regulate tumour angiogenesis.^24^ Next, we focus on the relationship between DKC1 and HIF-1α. HIF-1α is an active component of HIF-1 and belongs to the regulator of hypoxia response, thereby regulating a variety of physiological responses. Under hypoxic conditions, HIF-1α accumulates in the cytosol and is translocated to the nucleus, thereby forming active HIF-1α with HIF-1β and regulating downstream target genes transcriptionally.^21^ In this study, DKC1 can positively regulate HIF-1α in hypoxic and normoxic environments (Fig. 3a, b). DKC1 plays a transcriptional role in somatic cell reprogramming and stem cell maintenance. Fong et al.^47^ found that DKC1, which is the basic component of snoRNPs, regulates transcription initiation. Therefore, we speculated whether DKC1 directly acted on HIF-1α promoter to enhance the ability of CRC angiogenesis and metastasis. The result of ChIP-qRT-PCR investigated that DKC1 can bind to the P3, P4, P5 binding regions of HIF-1α promoter (Fig. 5e).

Subcutaneous tumour model also showed that DKC1 knockdown significantly inhibited tumour size. Therefore, we speculated whether DKC1 has an effect on CRC cell proliferation. We performed CCK8 analysis to detect the cell proliferation, and the result indicated that DKC1 knockdown significantly inhibited HCT116 and DLD1 cell proliferation. To verify this result further, we performed a flow cytometry to detect the change in cell cycle with DKC1 knockdown. Many studies have shown that the disorder of cell cycle can have a huge impact on cell proliferation.^48^ However, DKC1 knockdown did not seem to affect the cell cycle of HCT116 and DLD1 cells. In hypoxic conditions, the increase in the telomere length of mesenchymal stem cell can significantly stimulate mesenchymal stem cell proliferation^49^ and telomerase activity inhibition slowed cell proliferation in cervical cancer^50^ and glioma.^51^ Dyskerin is a key constituent of telomerase, and DKC1 maintains telomerase activity. Dyskerin inhibition effectively reduces telomerase activity has been confirmed in neuroblastoma.^29^ In future research, we will focus on whether DKC1 affects CRC proliferation by affecting telomerase activity.

In conclusion, our study demonstrated for the first time the role of DKC1 in CRC angiogenesis and metastasis. The results showed that DKC1 directly bound to the promoter region of HIF-1α to enhance HIF-1α transcription, increase HIF-1α and VEGF expression, and promote CRC progression. Therefore, DKC1 may be judged as an accurate indicator in predicting the prognosis of patients with CRC and can be acted as a potential therapeutic target for CRC.

## Supplementary information

SUPPLEMENTAL Tables and Figures

## Data Availability

The datasets used and/or analysed during the current study are available from the corresponding author on reasonable request.

## References

[1] Bray, F., Ferlay, J., Soerjomataram, I., Siegel, R. L., Torre, L. A. & Jemal, A. Global cancer statistics 2018: GLOBOCAN estimates of incidence and mortality worldwide for 36 cancers in 185 countries. *CA Cancer J. Clin.* **68**, 394–424 (2018). 30207593 10.3322/caac.21492

[2] Markowitz, S. D. & Bertagnolli, M. M. Molecular origins of cancer: molecular basis of colorectal cancer. *N. Engl. J. Med.* **361**, 2449–2460 (2009). 20018966 10.1056/NEJMra0804588 PMC2843693

[3] Chen, W. Cancer statistics: updated cancer burden in China. *Chin. J. Cancer Res.* **27**, 1 (2015). 25717219 10.3978/j.issn.1000-9604.2015.02.07 PMC4329178

[4] Cummings, L. C., Payes, J. D. & Cooper, G. S. Survival after hepatic resection in metastatic colorectal cancer: a population-based study. *Cancer* **109**, 718–726 (2007). 17238180 10.1002/cncr.22448

[5] Navarro, M., Nicolas, A., Ferrandez, A. & Lanas, A. Colorectal cancer population screening programs worldwide in 2016: an update. *World J. Gastroenterol.* **23**, 3632–3642 (2017). 28611516 10.3748/wjg.v23.i20.3632 PMC5449420

[6] Brenner, H., Kloor, M. & Pox, C. P. Colorectal cancer. *Lancet* **383**, 1490–1502 (2014). 24225001 10.1016/S0140-6736(13)61649-9

[7] Gutman, A., Frumkin, A., Adam, A., Bloch-Shtacher, N. & Rozenszajn, L. A. X-linked dyskeratosis congenita with pancytopenia. *Arch. Dermatol.* **114**, 1667–1671 (1978). 568915

[8] Heiss, N. S., Knight, S. W., Vulliamy, T. J., Klauck, S. M., Wiemann, S., Mason, P. J. et al. X-linked dyskeratosis congenita is caused by mutations in a highly conserved gene with putative nucleolar functions. *Nat Genet* **19**, 32–38 (1998). 9590285 10.1038/ng0598-32

[9] Knight, S. W., Vulliamy, T., Forni, G. L., Oscier, D., Mason, P. J. & Dokal, I. Fine mapping of the dyskeratosis congenita locus in Xq28. *J. Med. Genet.* **33**, 993–995 (1996). 9004129 10.1136/jmg.33.12.993 PMC1050808

[10] Connor, J. M., Gatherer, D., Gray, F. C., Pirrit, L. A. & Affara, N. A. Assignment of the gene for dyskeratosis congenita to Xq28. *Hum. Genet*. **72**, 348–351 (1986). 3009302 10.1007/BF00290963

[11] Chang, J. T., Chen, Y. L., Yang, H. T., Chen, C. Y. & Cheng, A. J. Differential regulation of telomerase activity by six telomerase subunits. *Eur. J. Biochem.* **269**, 3442–3450 (2002). 12135483 10.1046/j.1432-1033.2002.03025.x

[12] Cohen, S. B., Graham, M. E., Lovrecz, G. O., Bache, N., Robinson, P. J. & Reddel, R. R. Protein composition of catalytically active human telomerase from immortal cells. *Science* **315**, 1850–1853 (2007). 17395830 10.1126/science.1138596

[13] Wong, J. M. & Collins, K. Telomerase RNA level limits telomere maintenance in X-linked dyskeratosis congenita. *Genes Dev.* **20**, 2848–2858 (2006). 17015423 10.1101/gad.1476206 PMC1619937

[14] Young, N. S. Telomere biology and telomere diseases: implications for practice and research. *Hematology Am. Soc. Hematol. Educ. Program* **2010**, 30–35 (2010). 21239767 10.1182/asheducation-2010.1.30 PMC6380489

[15] McCaul, J. A., Gordon, K. E., Clark, L. J. & Parkinson, E. K. Telomerase inhibition and the future management of head-and-neck cancer. *Lancet Oncol.* **3**, 280–288 (2002). 12067805 10.1016/s1470-2045(02)00729-5

[16] Tang, C. M. & Yu, J. Hypoxia-inducible factor-1 as a therapeutic target in cancer. *J. Gastroenterol. Hepatol.* **28**, 401–405 (2013). 23173651 10.1111/jgh.12038

[17] Xu, Y., Xu, J., Yang, Y., Zhu, L., Li, X. & Zhao, W. SRGN promotes colorectal cancer metastasis as a critical downstream target of HIF-1alpha. *Cell Physiol. Biochem.* **48**, 2429–2440 (2018). 30121667 10.1159/000492657

[18] Wang, G. L., Jiang, B. H., Rue, E. A. & Semenza, G. L. Hypoxia-inducible factor 1 is a basic-helix-loop-helix-PAS heterodimer regulated by cellular O2 tension. *Proc. Natl Acad. Sci. USA* **92**, 5510–5514 (1995). 7539918 10.1073/pnas.92.12.5510 PMC41725

[19] Jain, S., Dolwick, K. M., Schmidt, J. V. & Bradfield, C. A. Potent transactivation domains of the Ah receptor and the Ah receptor nuclear translocator map to their carboxyl termini. *J. Biol. Chem.* **269**, 31518–31524 (1994). 7989319

[20] Jiang, B. H., Semenza, G. L., Bauer, C. & Marti, H. H. Hypoxia-inducible factor 1 levels vary exponentially over a physiologically relevant range of O2 tension. *Am. J. Physiol.* **271**, C1172–C1180 (1996). 8897823 10.1152/ajpcell.1996.271.4.C1172

[21] Soni, S. & Padwad, Y. S. HIF-1 in cancer therapy: two decade long story of a transcription factor. *Acta Oncol*. **56**, 503–515 (2017). 28358664 10.1080/0284186X.2017.1301680

[22] Liao, D., Corle, C., Seagroves, T. N. & Johnson, R. S. Hypoxia-inducible factor-1alpha is a key regulator of metastasis in a transgenic model of cancer initiation and progression. *Cancer Res.* **67**, 563–572 (2007). 17234764 10.1158/0008-5472.CAN-06-2701

[23] Chen, Y., Zhang, B., Bao, L., Jin, L., Yang, M., Peng, Y. et al. ZMYND8 acetylation mediates HIF-dependent breast cancer progression and metastasis. *J. Clin. Invest.* **128**, 1937–1955 (2018). 29629903 10.1172/JCI95089 PMC5919820

[24] Ciccone, V., Terzuoli, E., Donnini, S., Giachetti, A., Morbidelli, L. & Ziche, M. Stemness marker ALDH1A1 promotes tumor angiogenesis via retinoic acid/HIF-1alpha/VEGF signalling in MCF-7 breast cancer cells. *J. Exp. Clin. Cancer Res.* **37**, 311 (2018). 30541574 10.1186/s13046-018-0975-0 PMC6291966

[25] Dang, D. T., Chen, F., Gardner, L. B., Cummins, J. M., Rago, C., Bunz, F. et al. Hypoxia-inducible factor-1alpha promotes nonhypoxia-mediated proliferation in colon cancer cells and xenografts. *Cancer Res.* **66**, 1684–1936 (2006). 16452228 10.1158/0008-5472.CAN-05-2887

[26] Soung, Y. H., Lee, J. W., Kim, S. Y., Nam, S. W., Park, W. S., Lee, J. Y. et al. Absence of DKC1 exon 3 mutation in common human cancers. *Acta Oncol.* **45**, 342–343 (2006). 16644581 10.1080/02841860500437336

[27] Sieron, P., Hader, C., Hatina, J., Engers, R., Wlazlinski, A., Muller, M. et al. DKC1 overexpression associated with prostate cancer progression. *Br. J. Cancer* **101**, 1410–1416 (2009). 19755982 10.1038/sj.bjc.6605299 PMC2768451

[28] Liu, B., Zhang, J., Huang, C. & Liu, H. Dyskerin overexpression in human hepatocellular carcinoma is associated with advanced clinical stage and poor patient prognosis. *PLoS One* **7**, e43147 (2012). 22912812 10.1371/journal.pone.0043147 PMC3418259

[29] O'Brien, R., Tran, S. L., Maritz, M. F., Liu, B., Kong, C. F., Purgato, S. et al. MYC-driven neuroblastomas are addicted to a telomerase-independent function of dyskerin. *Cancer Res*. **76**, 3604–3617 (2016). 27197171 10.1158/0008-5472.CAN-15-0879

[30] Bellodi, C., Krasnykh, O., Haynes, N., Theodoropoulou, M., Peng, G., Montanaro, L. et al. Loss of function of the tumor suppressor DKC1 perturbs p27 translation control and contributes to pituitary tumorigenesis. *Cancer Res.* **70**, 6026–6035 (2010). 20587522 10.1158/0008-5472.CAN-09-4730 PMC2913864

[31] Shi, M., Cao, M., Song, J., Liu, Q., Li, H., Meng, F. et al. PinX1 inhibits the invasion and metastasis of human breast cancer via suppressing NF-kappaB/MMP-9 signaling pathway. *Mol. Cancer* **14**, 66 (2015). 25888829 10.1186/s12943-015-0332-2 PMC4404090

[32] Hu, Y. D., Yu, K. K., Wang, G., Zhang, D. P., Shi, C. J., Ding, Y. H. et al. Lanatoside C inhibits cell proliferation and induces apoptosis through attenuating Wnt/beta-catenin/c-Myc signaling pathway in human gastric cancer cell. *Biochem. Pharmacol.* **150**, 278–290 (2018). 10.1016/j.bcp.2018.02.023 29475060

[33] Zhou, X. P., Xie, S., Wu, S. S., Qi, Y. H., Wang, Z. H., Zhang, H. et al. Golgi phosphoprotein 3 promotes glioma progression via inhibiting Rab5-mediated endocytosis and degradation of epidermal growth factor receptor. *Neuro. Oncol.* **19**, 1628–1639 (2017). 28575494 10.1093/neuonc/nox104 PMC5716177

[34] Zhou, X. Y., Zhao, Y., Wang, J., Wang, X., Chen, C. X., Yin, D. et al. Resveratrol represses estrogen-induced mammary carcinogenesis through NRF2-UGT1A8-estrogen metabolic axis activation. *Biochem. Pharmacol.* **155**, 252–263 (2018). 30009768 10.1016/j.bcp.2018.07.006

[35] Hou, P. F., Jiang, T., Chen, F., Shi, P. C., Li, H. Q., Bai, J. et al. KIF4A facilitates cell proliferation via induction of p21-mediated cell cycle progression and promotes metastasis in colorectal cancer. *Cell Death Dis.* **9**, 477 (2018). 29706624 10.1038/s41419-018-0550-9 PMC5924760

[36] Ji, S., Tang, S., Li, K., Li, Z., Liang, W., Qiao, X. et al. Licoricidin inhibits the growth of SW480 human colorectal adenocarcinoma cells in vitro and in vivo by inducing cycle arrest, apoptosis and autophagy. *Toxicol. Appl. Pharmacol.* **326**, 25–33 (2017). 28416456 10.1016/j.taap.2017.04.015

[37] Liu, X., Cai, W., Niu, M., Chong, Y., Liu, H., Hu, W. et al. Plumbagin induces growth inhibition of human glioma cells by downregulating the expression and activity of FOXM1. *J. Neurooncol.* **121**, 469–477 (2015). 25528634 10.1007/s11060-014-1664-2

[38] Rankin, E. B. & Giaccia, A. J. Hypoxic control of metastasis. *Science* **352**, 175–180 (2016). 27124451 10.1126/science.aaf4405 PMC4898055

[39] Kienle, P., Koch, M., Autschbach, F., Benner, A., Treiber, M., Wannenmacher, M. et al. Decreased detection rate of disseminated tumor cells of rectal cancer patients after preoperative chemoradiation: a first step towards a molecular surrogate marker for neoadjuvant treatment in colorectal cancer. *Ann. Surg.* **238**, 324–330 (2003). discussion 330–321. 14501498 10.1097/01.sla.0000086547.27615.e6 PMC1422712

[40] Colon Cancer Laparoscopic or Open Resection Study G, Buunen, M., Veldkamp, R., Hop, W. C., Kuhry, E., Jeekel, J. et al. Survival after laparoscopic surgery versus open surgery for colon cancer: long-term outcome of a randomised clinical trial. *Lancet Oncol*. **10**, 44–52 (2009). 19071061 10.1016/S1470-2045(08)70310-3

[41] Kuellmer, A., Mueller, J., Caca, K., Aepli, P., Albers, D., Schumacher, B. et al. Endoscopic full-thickness resection for early colorectal cancer. *Gastrointest. Endosc.* 89, 1180–1189 (2019). 10.1016/j.gie.2018.12.025 30653939

[42] Fok, W. C., Niero, E. L. O., Dege, C., Brenner, K. A., Sturgeon, C. M. & Batista, L. F. Z. p53 Mediates failure of human definitive hematopoiesis in dyskeratosis congenita. *Stem Cell Reports* **9**, 409–418 (2017). 28757166 10.1016/j.stemcr.2017.06.015 PMC5550027

[43] Zhang, M., Pan, Y., Jiang, R., Hou, P., Shan, H., Chen, F. et al. DKC1 serves as a potential prognostic biomarker for human clear cell renal cell carcinoma and promotes its proliferation, migration and invasion via the NFkappaB pathway. *Oncol. Rep.* **40**, 968–978 (2018). 29901172 10.3892/or.2018.6484

[44] Carmeliet, P. Angiogenesis in life, disease and medicine. *Nature* **438**, 932–936 (2005). 16355210 10.1038/nature04478

[45] Folkman, J. Role of angiogenesis in tumor growth and metastasis. *Semin. Oncol.* **29**, 15–18 (2002). 12516034 10.1053/sonc.2002.37263

[46] Itakura, J., Ishiwata, T., Shen, B., Kornmann, M. & Korc, M. Concomitant over-expression of vascular endothelial growth factor and its receptors in pancreatic cancer. *Int J Cancer* **85**, 27–34 (2000). 10585578 10.1002/(sici)1097-0215(20000101)85:1<27::aid-ijc5>3.0.co;2-8

[47] Fong, Y. W., Ho, J. J., Inouye, C. & Tjian, R. The dyskerin ribonucleoprotein complex as an OCT4/SOX2 coactivator in embryonic stem cells. *Elife*, **3**, e03573 (2014). 10.7554/eLife.03573 PMC4270071 25407680

[48] Kramer, H. B., Lai, C. F., Patel, H., Periyasamy, M., Lin, M. L., Feller, S. M. et al. LRH-1 drives colon cancer cell growth by repressing the expression of the CDKN1A gene in a p53-dependent manner. *Nucleic Acids Res.* **44**, 582–594 (2016). 26400164 10.1093/nar/gkv948 PMC4737183

[49] Roychowdhury, S. & Talpaz, M. Managing resistance in chronic myeloid leukemia. *Blood Rev.* **25**, 279–290 (2011). 21982419 10.1016/j.blre.2011.09.001

[50] Feng, J., Funk, W. D., Wang, S. S., Weinrich, S. L., Avilion, A. A., Chiu, C. P. et al. The RNA component of human telomerase. *Science* **269**, 1236–1241 (1995). 7544491 10.1126/science.7544491

[51] Mata, J. E., Joshi, S. S., Palen, B., Pirruccello, S. J., Jackson, J. D., Elias, N. et al. A hexameric phosphorothioate oligonucleotide telomerase inhibitor arrests growth of Burkitt's lymphoma cells in vitro and in vivo. *Toxicol. Appl. Pharmacol.* **144**, 189–197 (1997). 9169084 10.1006/taap.1997.8103

