# Supplementary material for: DKC1 enhances angiogenesis by promoting HIF-1α transcription and facilitates metastasis in colorectal cancer
Source: Br J Cancer. 2019 Dec 20;122(5):668–79. doi: 10.1038/s41416-019-0695-z (PMC7054532; doi:10.1038/s41416-019-0695-z)
Supplement: Supplementary file 1 — SUPPLEMENTAL Tables and Figures [file 41416_2019_695_MOESM1_ESM.docx]

| **Supplementary Table S1 Univariate Cox regression analysis of DKC1 expression and clinicopathologic variables predicting the survival of CRC patients** | | | | | | | | | | | | | |
| --- | --- | --- | --- | --- | --- | --- | --- | --- | --- | --- | --- | --- | --- |
| **Variables^a^** | | | **Overall survival** | | | | |  | **Disease-speciﬁc survival** | | | | |
|  |  |  |  |  |  |  |  |  |  |  |  |  |  |
|  |  |  | **HR (95% CI)** | | ***P*** | | |  | **HR (95% CI)** | | ***P*** | | |
| **DKC1** | | |  | |  | | |  |  | | |  | |
| low | | |  | |  | | |  |  | | |  | |
| high | | | 1.928 (1.296-2.868) | | 0.001 | | |  | 2.023(1.349-3.035) | | | 0.001 | |
| **Age** | | |  | |  | | |  |  | | |  | |
| ≤60 | | |  | |  | | |  |  | | |  | |
| ＞60 | | | 1.297 (0.897-1.874) | | 0.167 | | |  | 1.249(0.854-1.827) | | | 0.252 | |
| **Gender** | | |  | |  | | |  |  | | |  | |
| Males | | |  | |  | | |  |  | | |  | |
| Females | | | 1.412 (0.985-2.023) | | 0.068 | | |  | 1.386(0.957-2.008) | | | 0.084 | |
| **Differentiate** | | |  | |  | | |  |  | | |  | |
| Poor | | |  | |  | | |  |  | | |  | |
| Moderate/high | | | 0.691 (0.464-1.029) | | 0.069 | | |  | 0.616(0.411-0.923) | | | 0.019 | |
| **Depth of invasion** | | |  | |  | | |  |  | | |  | |
| T1/T2 | | |  | |  | | |  |  | | |  | |
| T3/T4 | | | 1.387 (0.937-2.054) | | 0.102 | | |  | 1.274(0.853-1.902) | | | 0.236 | |
| **TNM stage** | | |  | |  | | |  |  | | |  | |
| I | | |  | |  | | |  |  | | |  | |
| II | | | 4.964 (2.444-10.081) | | <0.001 | | |  | 5.447(2.589-11.456) | | | <0.001 | |
| III | | | 7.217 (3.556-14.646) | | <0.001 | | |  | 7.183(3.391-15.217) | | | <0.001 | |
| IV | | | 11.325 (3.461-37.056) | | <0.001 | | |  | 11.169(2.941-42.417) | | | <0.001 | |
| **LNM** | | |  | |  | | |  |  | | |  | |
| N0 | | |  | |  | | |  |  | | |  | |
| N1/N2/N3 | | | 1.979 (1.387-2.823) | | <0.001 | | |  | 1.792(1.242-2.584) | | | 0.002 | |
| **Tumor diameter** | | |  | |  | | |  |  | | |  | |
| ≤5cm | | |  | |  | | |  |  | | |  | |
| ＞5cm | | | 1.177 (0.782-1.771) | | 0.435 | | |  | 1.098(0.717-1.683) | | | 0.667 | |
| **Distant metastasis** | | |  | |  | | |  |  | | |  | |
| M0 | | |  | |  | | |  |  | | |  | |
| M1 | | | 1.066 (0.496-2.290) | | 0.87 | | |  |  | | |  | |
| ***HR hazard ratio, CI conﬁdence interval, LNM lymph node metastasis*** | | | | | | | | | | | | | |
| **Supplementary Table S2 Multivariate Cox regression analysis models assessing the effects of covariates on overall and disease-free survival in CRC patients** | | | | | | | | | | | |  |  |
| **Variables^a^** | **Overall survival** | | | |  | **Disease-free survival** | | | | | |  |  |
|  | **HR (95% CI)** | | ***P*** | |  | **HR (95% CI)** | | | ***P*** | | |  |  |
| DKC1 |  | |  | |  |  | | |  | | |  |  |
| low |  | |  | |  |  | | |  | | |  |  |
| high | 1.556(1.016-2.383) | | 0.042 | |  | 1.655(1.071-2.588) | | | 0.023 | | |  |  |
| Age |  | |  | |  |  | | |  | | |  |  |
| ≤60 |  | |  | |  |  | | |  | | |  |  |
| ＞60 | 0.712(0.450-1.128) | | 0.148 | |  | 0.650(0.406-1.039) | | | 0.072 | | |  |  |
| Gender |  | |  | |  |  | | |  | | |  |  |
| Males |  | |  | |  |  | | |  | | |  |  |
| Females | 1.299(0.893-1.888) | | 0.172 | |  | 1.267(0.865-1.855) | | | 0.225 | | |  |  |
| Differentiate |  | |  | |  |  | | |  | | |  |  |
| Poor |  | |  | |  |  | | |  | | |  |  |
| Moderate/high | 0.757(0.501-1.145) | | 0.188 | |  | 0.713(0.469-1.085) | | | 0.114 | | |  |  |
| Depth of invasion | | |  | |  |  | | |  | | |  |  |
| T1/T2 |  | |  | |  |  | | |  | | |  |  |
| T3/T4 | 1.202(0.793-1.823) | | 0.385 | |  | 1.135(0.742-1.735) | | | 0.559 | | |  |  |
| TNM stage |  | |  | |  |  | | |  | | |  |  |
| I |  | |  | |  |  | | |  | | |  |  |
| II | 6.192(2.773-13.826) | | <0.001 | |  | 7.099(3.084-16.340) | | | <0.001 | | |  |  |
| III | 5.947(2.615-13.526) | | <0.001 | |  | 5.904(2.502-13.930) | | | <0.001 | | |  |  |
| IV | 9.686(2.538-36.965) | | 0.001 | |  | 10.74(2.453-47.012) | | | 0.002 | | |  |  |
| LNM |  | |  | |  |  | | |  | | |  |  |
| N0 |  | |  | |  |  | | |  | | |  |  |
| N1/N2/N3 | 1.250(0.737-2.119) | | 0.407 | |  | 1.248 (0.734-2.125) | | | 0.413 | | |  |  |

***HR hazard ratio, CI conﬁdence interval, LNM lymph node metastasis***


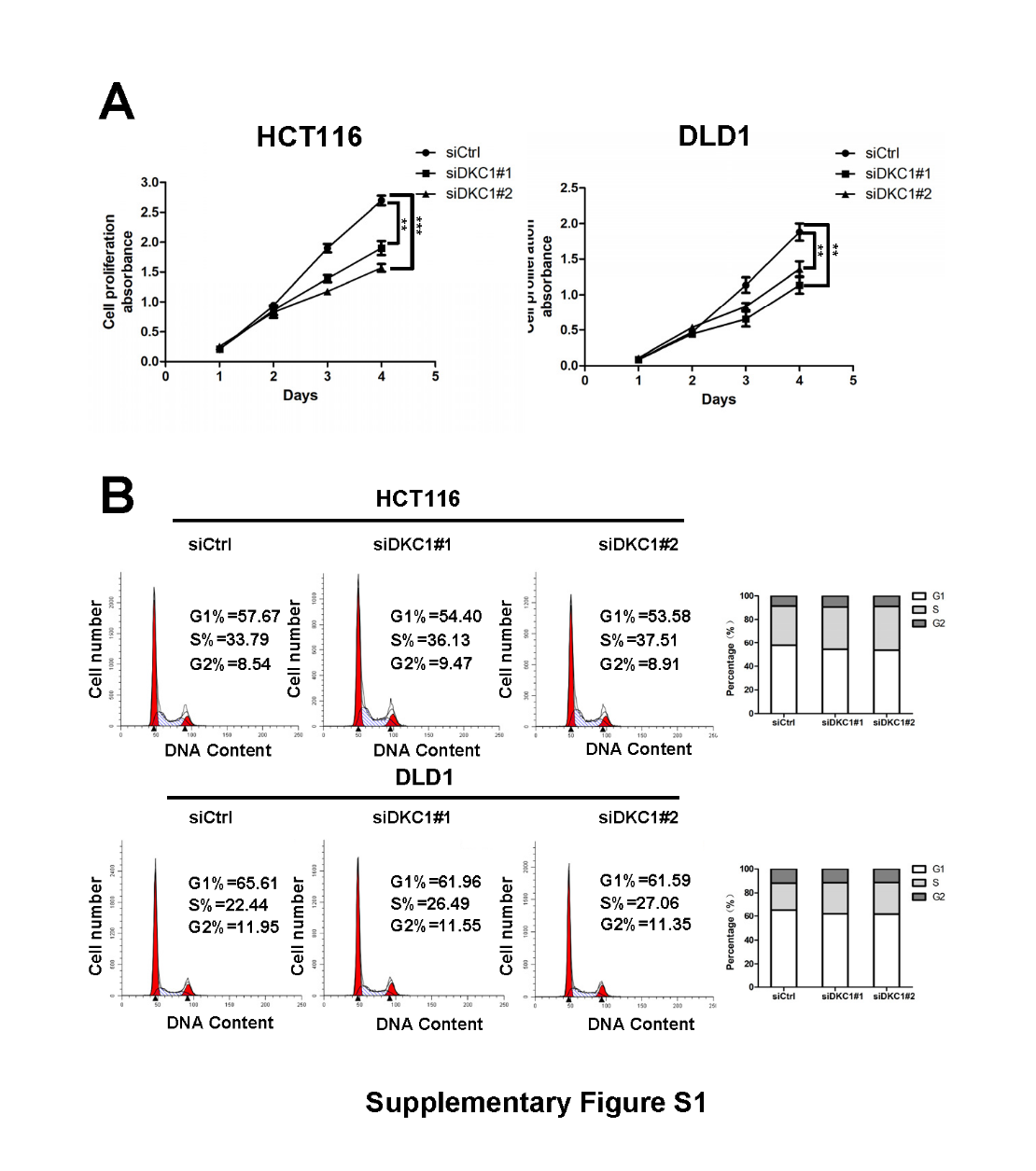


**Supplementary Figure S1: DKC1 facilitates cell proliferation but has no effect on cell cycle.** (A) Knockdown of DKC1 reduces the proliferation rate of HCT116 and DLD1 cells. (B) Knockdown of DKC1 has no effect on cell cycle as detected by ﬂow cytometric analysis following PI staining.
